# Supplementary material for: A hitchhiker's guide to deep chemical language processing for bioactivity prediction
Source: Digit Discov. 2024 Dec 16;4(2):316–25. doi: 10.1039/d4dd00311j (PMC11667676; doi:10.1039/d4dd00311j)
Supplement: DD-004-D4DD00311J-s001 [file DD-004-D4DD00311J-s001.pdf]

Supplementary Information (SI) for Digital Discovery.
This journal is © The Royal Society of Chemistry 2024

Supporting Information

A Hitchhiker's Guide to Deep Chemical Language Processing for Bioactivity Prediction

Rıza Özçelik and Francesca Grisoni

Table of Contents

Recall and root mean square error 2
Statistical testing 3

Recall and root mean square error

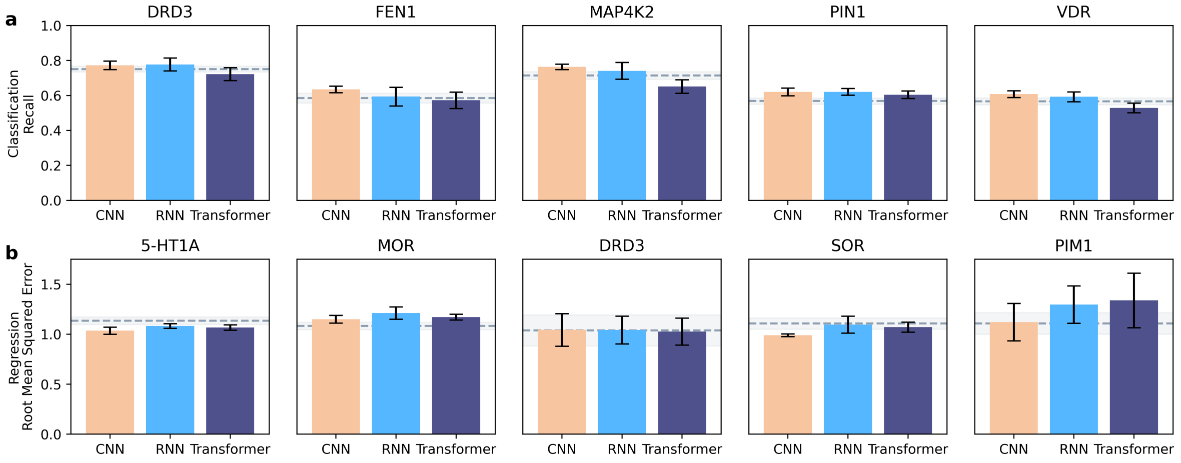

Supporting Figure S1. Recall and root mean square error of model architectures across datasets. These results were obtained with the same methodology as Figure 2.

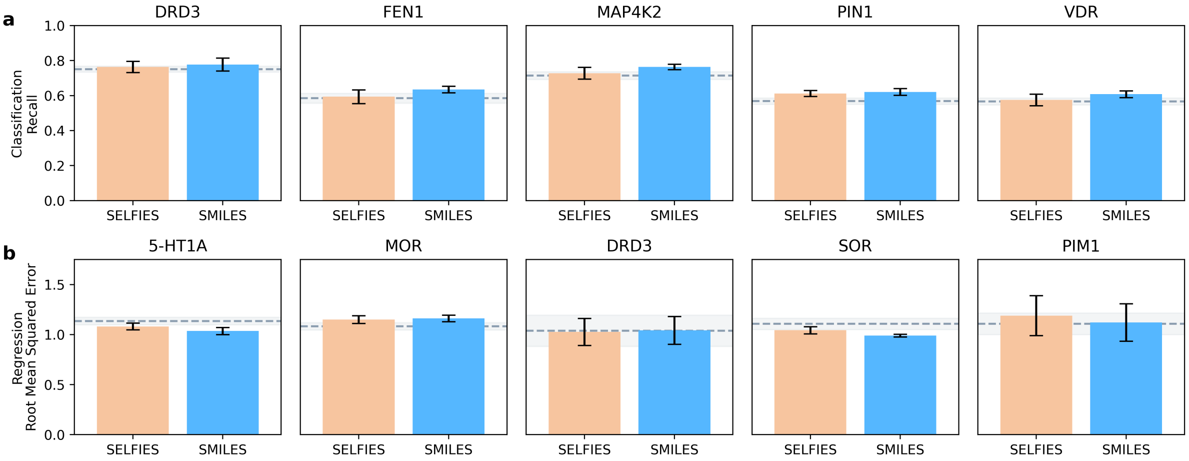

Supporting Figure S2. Recall and root mean square error of molecule representations across datasets. These results were obtained with the same methodology as Figure 3.

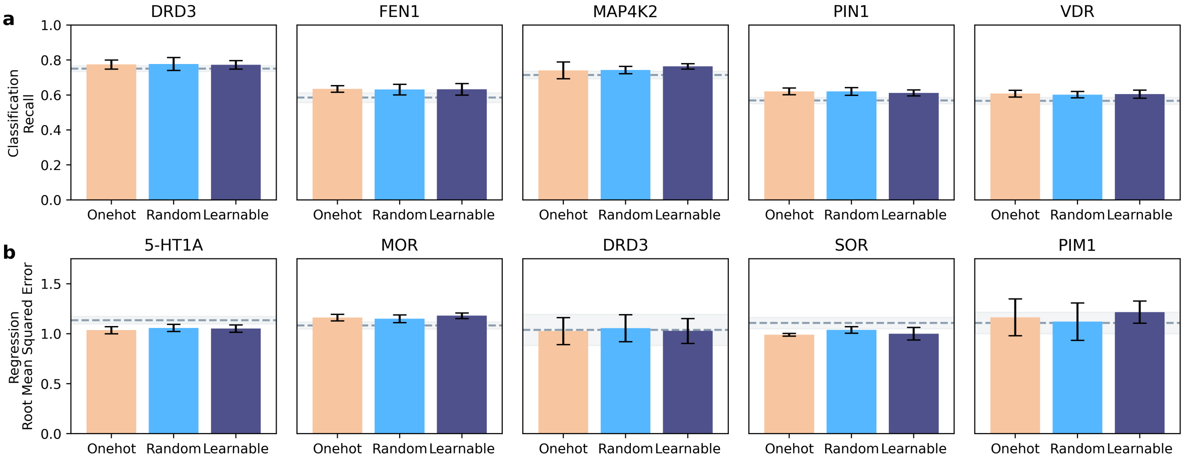

Supporting Figure S3. Recall and root mean squared error of molecule encodings across datasets. These results were obtained with the same methodology as Figure 3.

Statistical testing

Differences among methods.

Supporting Table S1. Results of the Friedman test for both classification and regression.

Friedman test	Classification	Regression
Statistic	32.28	2.75
p value	4.57E-07	0.43
N	25	25

Supporting Table S2. Post-hoc P values with Holm-Bonferroni correction

Architecture	CNN	GRU	Transformer	XGboost
CNN	*n.a.*	6.67E-02	6.41E-06	4.47E-06
GRU	6.67E-02	*n.a.*	3.25E-04	1.21E-02
Transformer	6.41E-06	3.25E-04	*n.a.*	9.80E-01
XGBoost	4.47E-06	1.21E-02	9.80E-01	*n.a.*

Differences between SMILES and SELFIES.

Regression: p-value=0.53
Classification: p-value=1.7E-03

Friedman test statistic:, p-value:
